# Use of Cystatin C and Serum Creatinine for the Diagnosis of Contrast-Induced Nephropathy in Patients Undergoing Contrast-Enhanced Computed Tomography at an Oncology Centre

**DOI:** 10.1371/journal.pone.0122877

**Published:** 2015-05-11

**Authors:** Joao Italo Fortalesa Melo, Rubens Chojniak, Debora Helena Costa Silva, Jose Carlos Oliveira Junior, Almir Galvão Vieira Bitencourt, Diego Holanda Silva, Marcos Duarte Guimarães, Hernandes Cerqueira Souza Silva, Denis Guilherme Teixeira Dias, Winglison Carli Rodrigues, Ellen Luzia Brancucci, Barbara Martins Soares Cruz, Beatriz Nunes Schiavon, Juliana Luz Passos Argenton, Margareth Arrivabene Camporini, Adriana Zocchio

**Affiliations:** 1 Department of Imaging - AC Camargo Cancer Center, São Paulo-SP, Brazil; 2 Department of Clinical Pathology - AC Camargo Cancer Center, São Paulo-SP, Brazil; 3 Statistical Nucleos - Campinas University School of Medicine, Campinas-SP, Brazil; UNIFESP Federal University of São Paulo, BRAZIL

## Abstract

**Objective:**

Our aim was to assess renal function using as laboratory measurements serum creatinine and cystatin C concentrations before and after administration of low-osmolarity (nonionic) iodinated contrast medium in patients with cancer undergoing computed tomography (CT).

**Methods:**

This prospective study included 400 oncologic outpatients. Serum creatinine and cystatin C concentrations were measured before and 72 h after contrast administration. Glomerular filtration rates (GFRs) were estimated using serum creatinine–based [Modification of Diet in Renal Disease (MDRD) and Cockroft-Gault and cystatin C based (Larsson) equations. Exploratory data analysis was performed. The nonparametric Wilcoxon test was used to compare pre and post contrast of test results and estimated clearance. The confidence interval used in the analysis was 95%.

**Results:**

Compared with the pre-contrast values, the mean serum creatinine concentration was significantly higher and average GFRs estimated using MDRD and Cockcroft-Gault equations were significantly lower after the administration of contrast (p <0.001). It was also observed a significant increase after contrast in the concentration of Cystatin C (p = 0.015). In addition, a decrease in GFR estimated using the average Larsson (p = 0.021) was observed between time points. However, none of the patients presented clinically significant nephropathy.

**Conclusions:**

Assessment using serum creatinine and cystatin C concentrations showed changes in renal function among patients with cancer undergoing contrast-enhanced CT examination in this study. No significant renal damage related to the use of low-osmolarity iodinated contrast medium of the type and dosage employed in this study was observed. This contrast medium is thus safe for use in patients with cancer.

## Introduction

An increasing number of patients undergo computed tomography (CT), and the use of iodinated contrast medium is necessary in most cases. CT is a widely used and often readily accessible tool of great importance, as it aids in the diagnosis of many conditions. However, the use of contrast medium is associated with risks, particularly in relation to renal function; this practice may be contributing to an increased incidence of contrast-induced nephropathy (CIN), which causes acute kidney injury (AKI) [1].

Exposure to contrast medium causes a substantial proportion of hospital-acquired AKI cases [2]. CIN was first described in the 1970s as the third leading cause of this complication, surpassed only by surgery and low blood pressure during hospitalisation [3][4]. The incidence of CIN-induced AKI has remained similar for more than two decades [5].

Based on observations of peak serum creatinine concentrations [8][9][10], CIN is classically defined as an increase in the serum creatinine concentration of 0.5 mg/dl or ≥25% from baseline within 48–72 h after the administration of iodinated contrast medium [11][6][12][13][14]. However, serum creatinine alone is not an accurate marker for the diagnosis of AKI because its extrarenal excretion can be affected by factors such as ethnicity, age, and protein ingestion [15][16][17]. Furthermore, recent studies have demonstrated fluctuations in serum creatinine concentration in patients not exposed iodinated contrast medium [7][18][19]. These factors indicate that the development of a more accurate method of diagnosing CIN-induced AKI is needed.

The use of cystatin C, a member of the cysteine proteinase inhibitor protein (cystatin C) superfamily, as a marker of renal function was proposed in 1985, but its routine laboratory use was only recently evaluated systematically. Cystatin C is a reliable biochemical marker of glomerular filtration due to its small size and high isoelectric point, which enable the filtering of this protein through the glomerular membrane and the reabsorption of a substantial proportion in the proximal tubule, where it is almost entirely catabolised. No non-renal pathway is known to be capable of eliminating cystatin C, and factors such as inflammatory and infectious processes do not alter the level of this protein, which depends primarily on glomerular filtration [20][21][22–27]. Cystatin C is more sensitive than serum creatinine to acute changes in kidney function and may be useful for the rapid detection of such changes. Preliminary data have suggested that cystatin C levels peak 24 h after exposure to contrast media, but few data on variations in these levels or the superiority of cystatin C to serum creatinine in predicting subsequent major events are available [28][29][30][31].

Contrast agents dose and type may be related to CIN. In addition, patients who have some factors including salt depletion, dehydration, anemia, age above 70 years, cardiovascular diseases, diabetes mellitus, smoking, and concurrent use of nephrotoxic drugs have more risk of CIN. Hyperlipidemia and alcohol consumption may also be associated with CIN [32,33]. Cancer patients usually have one or more of these factors, which increases the risk for renal impairment after contrast administration [34]. Few studies to date have evaluated the incidence of CIN and the use of contrast media for routine outpatient CT procedures (for monitoring, treatment, and/or restaging purposes) in patients with cancer who have undergone chemotherapy, radiotherapy, and/or surgery [35][36][37][38]. Studies have documented an incidence of chronic renal failure around for 6,7% in patients affected by malignancies, including those receiving dialysis [39]. Although contrast-enhanced CT is an essential tool in oncology, these factors cannot be ignored, particularly given the general brittleness of patients with cancer. The safety of clinical practices for this population should be a priority. The aim of this study was to evaluate the incidence of CIN in cancer outpatients after the administration of low-osmolarity iodinated contrast media. For a more accurate evaluation of renal function, both serum creatinine and cystatin C methods were used.

## Patients and Methods

### 2.1. Study population

This study was submitted to the Ethics Committee in Research of the Hospital AC Camargo Cancer Center and approved on May 30, 2011 with the number 1549/11. The written consent form was delivered in writing to all patients by researchers who also gave clarifications to all participants when they were unable to understand the consent form, only the legal responsible could sign for them. All children under 18 years of age who were included had the consent obtained by researchers of their legal responsible, no patient was included in the research without signing consent in writing. This prospective study involved 400 outpatients with cancer who underwent CT after the injection of low-osmolarity iodinated contrast at the AC Camargo Center, São Paulo, Brazil, between January 2012 and September 2013. Patients of any age and gender were considered for inclusion in the study. Pregnant women, patients with histories of renal failure and haemodialysis or peritoneal dialysis, those who had undergone more than one contrast-enhanced CT examination in the same week, and those receiving CIN prophylaxis were excluded. All participants met the imaging department’s selection criteria, which were designed to identify conditions favourable for the injection of contrast medium. The serum creatinine concentration < 1.5 mg/dL and no history of allergic reaction to contrast medium were considered to indicate suitability for the examination. Data on cancer type, histological grade, location, stage, metastasis, and treatment, as well as the receipt of chemotherapy prior to or concurrent with contrast-enhanced CT, were collected.

### 2.2. CT examination and laboratory testing

All tests and CT examinations were performed in the laboratory and imaging department, respectively, of the AC Camargo Cancer Center. All contrast-enhanced CT examinations were requested by the patients’ physicians for routine purposes, such as staging, follow up, or monitoring of disease evolution or remission. No CT examination was performed only for research purposes. Examinations were performed according to the imaging department’s protocol, with no additional cost, volume, or risk related to the use of contrast medium associated with this study. The amount of organically bound low-osmolarity (non-ionic) iodinated contrast (702 mOsm/kg osmolarity, 37°C, 320 mg/ml concentration; Tyco/Mallincrodt) injected was determined by the patient’s weight (90–125 ml at 1 ml/kg).

Blood samples were collected for serum assays immediately before and 72 h after contrast administration, when patients were asked to return for this purpose. Serum concentrations of cystatin C and creatinine were determined by nephelometry and the kinetic Jaffe method, respectively, using automated equipment. Established reference values were used in this study (cystatin C, 0.6–1.0 mg/l; creatinine, 0.6–1.5 mg/dl). Glomerular filtration rates (GFRs) were estimated using the widely applied Cockcroft-Gault [CG; (140 - age) × weight (kg) / serum creatinine × 72 × (0.85 if female)], Modification of Diet in Renal Disease [MDRD; 175 × serum creatinine^−1.154^ × age^−0.203^ × (0.742 if female)], and Larsson (77.24 × cystatin C^−1.2623^) equations [40–42][43]. GFRs were interpreted with reference to the international standards proposed by the National Kidney Foundation of London (NKF 2002), in which were defined, five stages of chronic kidney disease, ranging from one to five, where one is the mildest form of the disease and five would be a more severe where hemodialysis is now indispensable to life [15]. For serum creatine adopt the recommendations of the European Society of Urogenital Radiology (ESUR) standard, which defines a 25% increase in baseline creatinine or 0.5 mg/dL [14]. Cystatin C was considered to increase ≥ 10% from baseline of this marker. These standards were used to identify CIN [22].

### 2.3. Statistical analyses

Exploratory data analyses, including the calculation of descriptive statistics, was performed. The nonparametric Wilcoxon test was used to compare pre- and post-contrast test results and estimated GFRs. The chi-squared test was used to compare pre- and post-contrast stages of kidney disease, as defined using GFRs. A 95% confidence interval was used for all analyses. The Minitab 14 software was used for analysis.

## Results

A total of 400 patients aged 14–86 years participated in this study; 227 (56.9%) patients were female. Patients’ demographic and clinical characteristics are described on Table 1. Thirty-one (7.8%) patients did not return for post-contrast blood collection and examination results for 10 (4%) patients were incomplete; these patients were excluded from the sample. Pre- and post-contrast creatinine concentrations were available for 359 patients, allowing GFR estimation using the CG and MDRD equations; due to the high cost of cystatin C assessment, GFR estimation using the Larsson equation was possible for only 269 patients.

**Table 1:** Demographic and clinical characteristics of the included patients (n = 400).

VARIABLES	RESULTS	
AGE (n = 400)—mean (range)	53,7 years (14–86 years)	
GENDER (n = 399)—n(%)		
Female	227 (56,9%)	
Male	172 (43,1%)	
CHEMOTHERAPY (n = 240)—n(%)		
No	72 (30,0%)	
Yes	168 (70,0%)	
RADIOTHERAPY (n = 238)—n(%)		
No	150 (63,0%)	
Yes	88 (37,0%)	
METASTASIS (n = 240)—n(%)		
No	102 (42,5%)	
Yes	138 (57,5%)	
SINGLE KIDNEY (n = 394)—n(%)		
No	365 (92,6%)	
Yes	29 (7,4%)	
HISTOLOGIC TYPE (MORE COMMONS)—n(%)		
Colon Adenocarcinoma	29 (12,1%)	
Breast Invasive Ductal Carcinoma	29 (12,1%)	
Prostate Adenocarcinoma	10 (4,2%)	
Rectal Adnocarcinoma	8 (3,4%)	
Lung Adenocarcinoma	7 (2,9%)	
Renal Carcinoma Cells	6 (2,5%)	
Gastric Adenocarcinoma	5 (2,1%)	

**Fig 1:**
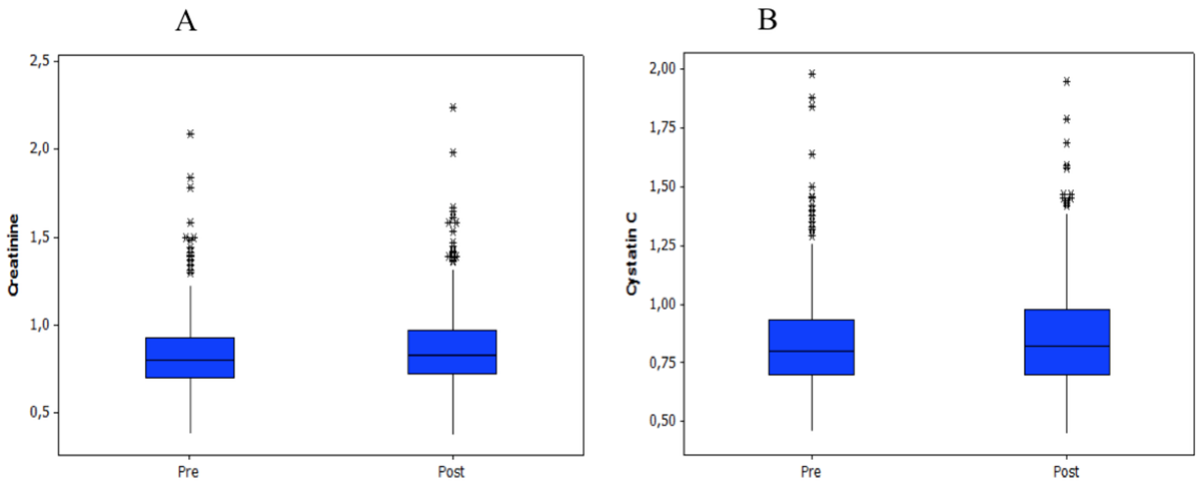
Box plots of serum creatinine (a) and cystatin C (b) concentrations before and after contrast administration.

**Fig 2:**
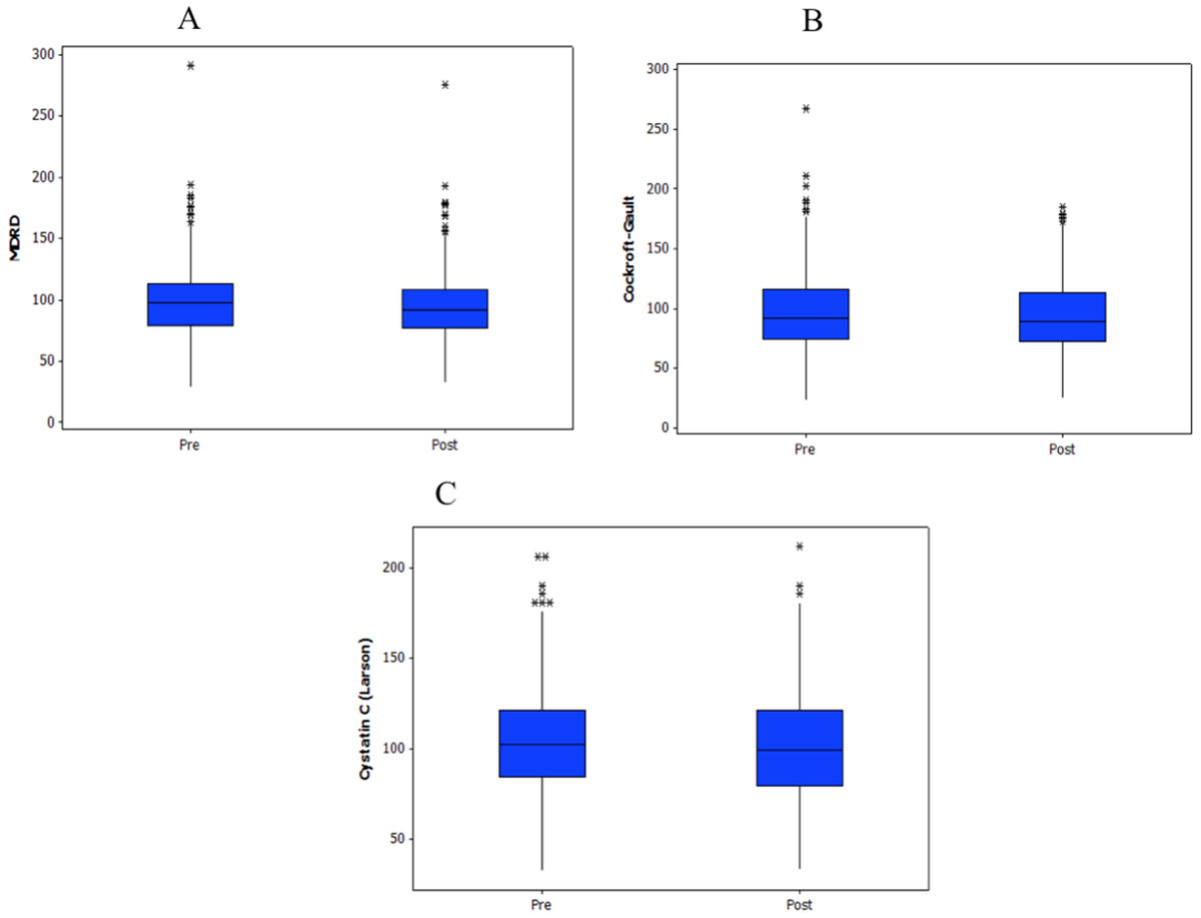
Box plots of pre- and post-contrast estimated glomerular filtration rates calculated using the Modification of Diet in Renal Disease MDRD (a), Cockroft-Gault (b), and Larsson (c) equations.

Compared with pre-contrast values, creatinine and cystatin C concentrations were significantly higher (*p* < 0.001 and *p* = 0.015, respectively) and estimated GFRs were significantly lower (CG and MDRD, *p* < 0.001; Larsson, *p* = 0.021) after contrast administration (Table 2). Box plots of these parameters (Figs 1 and 2) show several outlying values. Of 359 patients for whom pre- and post-contrast creatinine concentrations were obtained, CIN was identified in two (0.56%) patients [95% confidence interval (CI), 0.068–2.00%] based on a 0.5-mg/dl increase and in 33 (9.2%) patients (95% CI, 6.4–12.7%) based on a ≥25% increase from baseline serum creatinine concentrations. Seventy-five (27.9%; 95% CI, 22.6–33.7%) patients met the CIN criterion of ≥10% increase in cystatin C concentration [22]. However, only one patient had an increase of serum creatinine above the established reference values. None of the patients presented clinically significant CIN, which was defined as development of symptoms related to renal impairment or need of specific treatment such as dialysis to normalize renal function.

**Table 2:** Serum creatinine and cystatin C concentrations and estimated glomerular filtration rates before and after contrast administration.

Variable	*n* ^a^	Pre-contrast					Post-contrast					
		Mean	SD	Min	Med	Max	Mean	SD	Min	Med	Max	*p*
Creatinine (mg/dl)	359	0.846	0.226	0.380	0.800	2.090	0.881	0.237	0.370	0.830	2.240	**<0.001**
Cystatin C (mg/l)	269	0.849	0.238	0.460	0.800	1.980	0.866	0.242	0.450	0.820	1.950	**0.015**
eGFR (MDRD)	359	99.3	29.2	29.0	97.4	291.0	94.8	27.9	32.4	92.2	275.7	**<0.001**
eGFR (CG)	359	98.1	32.6	23.6	91.9	266.8	94.1	29.7	24.8	88.8	185.0	**<0.001**
eGFR (Larsson)	269	104.4	32.7	32.6	102.4	205.9	102.1	32.4	33.3	99.2	211.6	**0.021**

^a^Only patients for whom pre- and post-contrast test results were available were included in analyses.

SD = standard deviation, Min = minimum, Med = median, Max = maximum, eGFR = estimated glomerular filtration rate, MDRD = Modification of Diet in Renal Disease, CG = Cockroft-Gault.

Table 3 shows the distribution of pre- and post-contrast estimated GFRs according to the stages of kidney disease defined in the NKF 2002 international standards.

**Table 3:** Frequencies and percentages of patients in each stage of kidney disease before and after contrast administration, as determined by estimated glomerular filtration rates.

Equation	Timepoint	Kidney disease stage						
		1	2	3	4	5	2–5	*p*
		(GFR ≥ 90)	(GFR 60–89)	(GFR 30–59)	(GFR 15–29)	(GFR < 15 or dialysis)	(GFR < 90)	(1 *vs*. 2–5)
MDRD (*n* = 359)	Pre-contrast	221 (61.6)	119 (33.1)	18 (5.0)	1 (0.3)	0 (0)	138 (38.4)	
	Post-contrast	190 (52.9)	140 (39.0)	29 (8.1)	0 (0)	0 (0)	169 (47.1)	0.019
CG (*n* = 359)	Pre-contrast	192 (53.5)	139 (38.7)	26 (7.2)	2 (0.6)	0 (0)	167 (46.5)	
	Post-contrast	172 (47.9)	152 (42.3)	33 (9.2)	2 (0.6)	0 (0)	187 (52.1)	0.135
Larsson (*n* = 269)	Pre-contrast	179 (66.5)	74 (27.5)	16 (5.9)	0 (0)	0 (0)	90 (33.5)	
	Post-contrast	163 (60.6)	84 (31.2)	22 (8.2)	0 (0)	0 (0)	106 (39.4)	0.151

Data are presented as *n* (%). GFRs are in ml/min/1.73m^2^.

Stage 1 = kidney injury with normal or increased GFR, 2 = slight decrease in GFR, 3 = moderate decrease in GFR, 4 = severe decrease in GFR, 5 = renal failure.

GFR = glomerular filtration rate, MDRD = Modification of Diet in Renal Disease, CG = Cockroft-Gault.

## Discussion

In this study evaluating cancer patients undergoing CT with the intravenous injection of low-osmolarity (non-ionic) iodinated contrast, we observed an overall increase in serum creatinine and cystatin C values after contrast administration. The incidence of CIN was 9.2% when the serum creatinine criterion was used, and 27.9% when the cystatin C criterion was used, which was expected because the last is a more sensitive marker for kidney injury. However, only one patient had an increase on serum creatinine above the reference value and none presented clinically significant CIN.

The pathophysiology of CIN is not fully understood, but may involve haemodynamic changes, endothelial vasoactive mediators, aetiological factors, generation of free radicals, or direct tubular toxicity [44,45]. The incidence of CIN is approximately 2% in the general population [10], but it can reach 90% in high-risk patients [11]. Pre-existing risk factors complicating renal damage may include chronic underlying diseases such as diabetes mellitus, the type and volume of contrast medium, and the duration of treatment with anti-cancer drugs [46,47][37]. We also observed reductions in GFRs calculated using the MDRD and CG equations in association with the elevation of serum creatinine concentrations.

In an evaluation of 11,588 patients, Bruce et al. [7] observed that the incidence of creatinine elevation in control subjects undergoing unenhanced CT was statistically similar to that in patients undergoing CT with low-osmolar or isoosmolar contrast medium. Newhouse et al. [18] evaluated 32,161 patients who had not received contrast material and reported that more than half of these patients showed an increase of at least 25% in serum creatinine concentration and more than two-fifths showed an increase of at least 0.4 mg/dl. Briguori et al. [23] evaluated the incidence of CIN in 410 consecutive patients with chronic kidney disease undergoing either coronary and/or peripheral angiography and/or angioplasty, and found an increase ≥0.3mg/dl in serum creatinine on 8.2% and an increase ≥10% in serum cystatin C on 21.2%. We observed an increase ≥10% in serum cystatin C in 27.9% of our patients, however they developed no complication indicating the presence of CIN.

## Conclusions

Assessment using serum creatinine and cystatin C concentrations showed that there is a mild impairment in renal function among patients with cancer undergoing contrast-enhanced CT examination, however, the variation in laboratory measurements occurred within the normal reference values in almost all patients and no cases of clinically significant CIN was reported in our sample. These findings indicate that the use of low-osmolarity (non-ionic) iodinated contrast at the dosage employed in this study is safe in patients with cancer.
